# The Effect of Corrected Inflammation, Oxidative Stress and Endothelial Dysfunction on Fmd Levels in Patients with Selected Chronic Diseases: A Quasi-Experimental Study

**DOI:** 10.1038/s41598-020-65528-6

**Published:** 2020-06-02

**Authors:** Mahmut Ilker Yilmaz, Micol Romano, Mustafa Kemal Basarali, Abdelbaset Elzagallaai, Murat Karaman, Zeynep Demir, Muhammet Fatih Demir, Fatih Akcay, Melik Seyrek, Nuri Haksever, David Piskin, Rolando Cimaz, Michael J. Rieder, Erkan Demirkaya

**Affiliations:** 1 Epigenetic Health Solutions, Unit of Nephrology, Ankara, Turkey; 2 Division of Paediatric Rheumatology, Victoria Children’s Hospital, University of Western Ontario, London, ON Canada; 3 Department of Rheumatology, Istituto Ortopedico Gaetano Pini, Milano, Italy; 4 Ankara Provincial Health Directorate, Department of Biochemistry, Ankara, Turkey; 5 Physiology and Pharmacology, Schulich School of Medicine and Dentistry, University of Western Ontario, London, Canada; 6 Department of Paediatrics, Victoria Children’s Hospital, University of Western Ontario, London, Canada; 7 Medisante Health Clinic, Unit of Integrative Medicine, Ankara, Turkey; 8 Department of Endocrinology, Yeditepe University, Istanbul, Turkey; 9 Department of Clinical Sciences and Community Health, University of Milano, Milano, Italy

**Keywords:** Clinical trials, Cancer, Endocrine system and metabolic diseases, Neurological disorders, Rheumatic diseases

## Abstract

While the pathophysiology of chronic disorders varies there are three basic mechanisms - inflammation, oxidative stress and endothelial dysfunction – that are common in many chronic diseases. However, the failure of these mechanisms to work synchronously can lead to morbidity complicating the course of many chronic diseases. We analyzed data of 178 patients from cohorts with selected chronic diseases in this quasi-experimental study. Endothelial dysfunction was determined by flow-mediated dilatation (FMD) and asymmetric dimethylarginine (ADMA) levels. Serum ADMA, high sensitive C-reactive protein (hs-CRP), serum PTX3, malondialdehyde (MDA), Cu/Zn-superoxide dismutase (Cu/Zn-SOD), glutathione peroxidase (GSH-Px) levels and FMD were studied in baseline and after 12 weeks of Morinda citrifolia (anti-atherosclerotic liquid- AAL), omega-3 (anti-inflammatory capsules- AIC) and extract with Alaskan blueberry (anti-oxidant liquid- AOL). Stepwise multivariate regression analysis was used to evaluate the association of FMD with clinical and serologic parameters. Serum ADMA, MDA, PTX3, hsCRP and albumin levels, and proteinuria were significantly decreased while CuZn-SOD, GSH-Px and FMD levels were significantly increased following AAL, AIC and AOL therapies. The FMD was negatively correlated with serum ADMA, MDA, PTX3, and hsCRP levels and positively correlated with CuZn-SOD and eGFR levels. ADMA and PTX3 levels were independently related to FMD both before and after AAL, AIC and AOL therapies. Our study shows that serum ADMA, MDA, PTX3 levels are associated with endothelial dysfunction in patients with selected chronic diseases. In addition, short-term AAL, AIC and AOL therapies significantly improves a number of parameters in our cohort and can normalize ADMA, PTX3, hsCRP and MDA levels.

## Introduction

Chronic diseases are the leading causes of death in developed countries, with atherosclerosis and its complications at the top of the list. The commonest chronic diseases – cardiovascular disease, diabetes, obesity and Alzheimer’s Disease – cannot be prevented by disease or cured by medication, are more common in older patients and their health impacts are substantially worsened by health damaging behaviors such as tobacco use, lack of exercise and poor nutrition^1^.

Potential links between many chronic diseases include oxidative stress and inflammation. As an example, atherosclerosis is a chronic vascular disease characterized by inflammation, oxidative stress and endothelial dysfunction (ED). A key first step in the development of ED and the subsequent progression to atherosclerosis appears to be decreased bioavailability of nitric oxide. While risk factors associated with ED and atherosclerosis include hypertension and dyslipidemia, oxidative stress appears to the largest contributor to the formation of atherosclerotic plaques^2^. Oxidant stress and a perturbed balance between oxidants and antioxidant defense systems appears to be a common element in the pathophysiology of a number of diseases^3^. Endogenous defenses against oxidative injury include systems such as glutathione peroxidase (GSH-Px), superoxide dismutase (SOD), catalase, glutathione reductase, key trace elements and vitamins A, E and C^4^. When endogenous systems are unable to provide adequate antioxidant capacity oxygen radicals will cause lipid peroxidation and malondialdehyde (MDA) formation. Lipid peroxidation increases membrane permeability while MDA produces intra-and inter-molecular links that inactivate membrane transporters^5^, in both cases impeding key cellular functions^6,7^.

In addition to producing direct cellular injury, oxidant stress and ED can lead to inflammation^8^. Vascular endothelial inflammation and enhanced oxidative stress appear to be a key mechanism in accelerating the progression of atherosclerosis in the setting of chronic disease, as noted above adding to the disease burden of these disorders.

Thus, when taken in concert, inflammation, oxidative stress and endothelial dysfunction are linked factors that may be major contributors to the morbidity and mortality associated with a number of chronic diseases. This in turn suggests that approaches which modulate these factors may reduce the impact of chronic diseases on both quality of life and on premature loss of life.

Potential approaches include the development of anti-oxidant and anti-atherosclerotic compounds from food. *Morinda citrifolia* leaf, which is a dietary supplementation used in traditionally to prevent chronic diseases, may be effective as anti atherosclerotic agent. Noni was one of them and mostly used plants by Polynesian people for the treatment of diabetes^9^. Several *in vitro* and *in vivo* animal studies suggests noni fruit juice may have some effect as anticancer activity^10^.

Other approaches have included long-chain eicosapentaenoic acid (EPA) and docosahexaenoic acid (DHA) omega-3 fatty acid supplementation. Recent research suggests that supplemental long chain omega-3s have health benefits in conditions such as cardiovascular disease. There is some thought that plant omega-3 sources are nutritionally and therapeutically equivalent to the EPA/DHA omega-3 in fish oil. Clinical trials with DHA-rich oil have shown comparable efficacies to fish oil for protection from cardiovascular risk factors by lowering plasma triglycerides and oxidative stress^11^. There appears to be close interactions between the central nervous system, endocrine organs, cytokines, exercise, and dietary omega-3 fatty acids. This may explain why these fatty acids could be of benefit in the management of conditions as diverse as septicemia and septic shock, Alzheimer’s disease, Parkinson’s disease, inflammatory bowel diseases, diabetes mellitus, essential hypertension and atherosclerosis^12^.

Berry consumption has been suggested to be useful in cardiovascular disease prevention, although well conducted research on the prevention of atherosclerosis through consuming individual whole berries remains scarce. Therefore, further elucidating the role that berries play in the prevention of atherosclerosis is needed. From this perspective, blueberries were selected to articulate research strategies for studying atheroprotective effects of berries^13,14^.

In the present study, we aimed to pursue this question by testing the hypothesis that improvement in FMD in patients with chronic diseases whose pathophysiology involves oxidant stress and inflammation following initiation of anti-atherosclerotic liquid (AAL), anti-inflammatory capsules (AIC) and anti-oxidant liquid (AOL) therapies is directly linked to the relative reduction of inflammatory, oxidative stress and endothelial dysfunction markers, especially pentraxin 3 (PTX3), MDA and asymmetric dimethylarginine (ADMA).

## Material and Methods

### Participants

This study conducted with selected patients who referred to the Epigenetic Health Center Outpatient Clinics, Ankara, Turkey during the period December 1^st^, 2018 – June^1*st*^, 2019. This was a quasi-experimental pre and post-test designed study^15^. We followed “Improving the reporting quality of nonrandomized evaluations of behavioral and public health interventions: TREND” statement checklist^16^

We included patients who were: older than 18 years, with systolic blood pressure ≤140 mmHg and/or diastolic blood pressure ≤90 mmHg and a normal estimated glomerular filtration rate (eGFR) (≥90 mL/min). We excluded patients previously treated with an angiotensin converting enzyme inhibiters (ACEI) or angiotensin receptor blockers (ARBs), with obesity (BMI > 30 kg/m^2^), dyslipidemia (total cholesterol >280 mg/dl, fasting triglycerides >180 mg/dl), renal failure, (eGFR <90 ml/min), nephrotic syndrome (urinary protein excretion >3000 mg/day), a history of CVD (medical history, abnormal electrocardiogram, smoking and/or currently or within the last 3 months taking statins).

**Figure 1:**
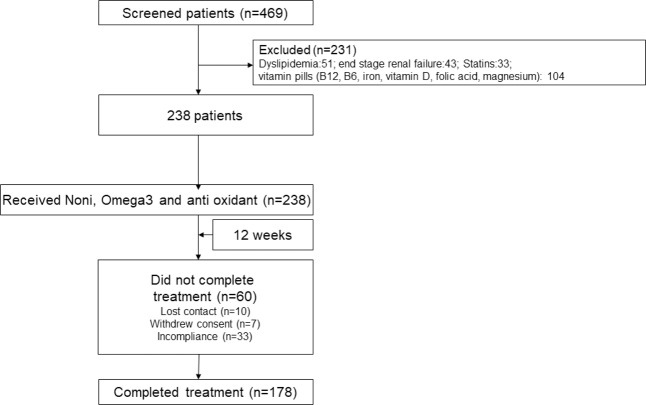
Flow chart of patients enrolled in the trial.

There were 469 patients who fulfilled the above inclusion criteria. A total of 178 patients (94 M, 58 ± 14 years) (Fig. 1) with chronic diseases including rheumatoid arthritis, familial Mediterranean fever (FMF), diabetes mellitus type-2, hypertension, multiple sclerosis, chronic obstructive pulmonary disease (COPD), Alzheimer disease, cancer was recruited to the study.

Study patients were evaluated by standard physical examination, chest X-ray, baseline electrocardiogram, two-dimensional echocardiography, and routine clinical laboratory tests, including liver and kidney function tests and 24-hour urinary protein measurements. Arterial blood pressure was measured in the right arm by mercury sphygmomanometer three times in a resting condition in the morning, and mean values were calculated for diastolic and systolic pressures.

### Intervention

In an open-label trial, patients were given a *Morinda citrifolia* (anti-atherosclerotic liquid- AAL, 3 ml once per day), omega-3 (anti-inflammatory capsules- AIC, 3 capsules once per day) and an extract of Alaskan blueberry and 21 different red purple fruit vegetables (anti-oxidant liquid- AOL, 30 ml once per day) therapies for 12 weeks immediately following baseline measurements. During the study period, serum creatinine and potassium concentrations were measured every 2 weeks and the dose of Noni, omega-3 and anti-oxidant therapies were titrated to achieve a serum potassium concentration <5.5 mEq/L. After this period, blood samples were obtained for measurements as shown below. All of these patients continue to receive their current treatment for their disease. None of these patients used either dietary supplements or any vitamin supplements.

### Measurements

#### Blood chemistry

Morning blood samples were collected from patients after 12 hours of fasting. Subjects were asked to refrain from physical activity for at least 30 minutes prior to the blood draw. In addition to routine clinical laboratory tests, serum ADMA, MDA, CuZn-SOD, GSH-Px, hsCRP and PTX3 concentrations and basal insulin levels were analyzed from all patients. After the intervention period, blood samples were obtained for the measurement of serum ADMA, MDA, CuZn-SOD, GSH-Px, hsCRP and PTX3 concentration. The measurement of total cholesterol (TC), triglyceride (TG), high-density lipoprotein (HDL) cholesterol and fasting plasma glucose (FPG) was performed by enzymatic colorimetric method with Olympus AU 600 auto analyzer using reagents from Olympus Diagnostics, GmbH (Hamburg, Germany). Low-density lipoprotein (LDL) cholesterol was calculated by Friedewald’s formula^17^.

Serum basal insulin values were determined by the coated tube method (DPC-USA). In particular, insulin resistances index Homeostasis Model Assessment-Insulin resistance (HOMA-IR) was computed with the formula: (HOMA-IR) = FPG (mg/dl) x immunoreactive insulin (IRI) (µIU/ml)/405^18^. All samples were run in triplicates.

#### ADMA measurements

Measurements of serum ADMA were done using high performance liquid chromatography (HPLC), as described by Chen *et al*.^19^. In brief, 20 mg of 5-sulfosalisilic acid (5-SSA) was added to 1 ml serum and the mixture was left in an ice-bath for 10 min. The precipitated protein was removed by centrifugation at 2000 g for 10 min. Ten micro liters of the supernatant which was filtered through a 0.2 µm filter was mixed with 100 µl of derivatization reagent (prepared by dissolving 10 mg o-phtaldialdehyde in 0.5 ml of methanol, 2 ml of 0.4 M borate buffer (pH 10.0) and 30 µl of 2-mercaptoethanol) and then injected into the chromatographic system. Separation of ADMA was achieved with a 150 × 4 mm I.D. Nova-pak C18 column with a particle size of 5 µm (Waters, Millipore, Milford, MA, USA) using 50 mM sodium acetate (pH 6.8), methanol and tetrahydrofurane as mobile phase (A, 82:17:1; B, 22:77:1) at a flow-rate of 1.0 ml/min. The area of peak detected by the fluorescent detector (Ex: 338 nm) was used as quantification. The variability of the method was less than 7%, and the detection limit of the assay was 0.01 µM.

#### High sensitive C reactive protein (hsCRP) assessment

Briefly, serum samples were diluted with a ratio of 1/101 with the diluents solution. Calibrators, kit controls and serum samples were all added on each micro well with an incubation period of 30 minutes. After 3 washing intervals 100 µL enzyme conjugate (peroxidase labeled anti-CRP) was added on each micro well for additional 15 minutes incubation in room temperature in dark. The reaction was stopped with a stop solution and photometric measurement was performed at the 450 nm wavelength^20^.

#### Plasma PTX-3 measurements

Plasma PTX 3 concentration was measured posteriori from frozen samples by using a commercially available enzyme-linked immunosorbent assay (ELISA) kit (Perseus Proteomics Inc, Japan).

#### Erythrocyte antioxidant capacity

Blood samples were drawn after overnight fasting from the antecubital vein and collected in heparinized polypropylene tubes. Plasma and erythrocytes were separated and used for measuring trace elements and antioxidant enzymes. Erythrocyte CuZn-SOD and GSH-Px activity was measured in a UV-VIS Recording Spectrophotometer (UV-2100S; Shimadzu Co., Kyoto, Japan) as previously described by Aydin *et al*.^21^. Erythrocyte zinc (Zn), copper (Cu), and iron (Fe) levels were measured by flame atomic absorption spectrophotometry using a Varian atomic absorption spectrophotometer (30/40 model; Varian Techtron Pty Ltd., Victoria, Australia). The wavelengths used were as follows: 213.9-nm wavelengths for Zn, 324.7-nm wavelengths for Cu, and 248.3-nm wavelengths for Fe. Results were expressed as units per milliliter for CuZn-SOD and GSH-Px and as micrograms per milliliter for Zn, Fe, and Cu.

Erythrocyte MDA level measurement: Erythrocyte MDA levels were determined on erythrocyte lysate obtained after centrifugation and in accordance with the method described by Jain^22^. After the reaction of thiobarbituric acid with MDA, the reaction product was measured spectrophotometrically at 532 nm. Tetrametoxypropane solution was used as standard. MDA levels of erythrocyte were expressed as nanomoles per milliliter.

#### Serum vitamin B12 measurement

Serum vitamin B12 was measured in adults 20 years and older using the fully automated electrochemiluminescence immunoassay on the Roche Elecsys 170 System (Roche Diagnostics, Indianapolis, IN). Vials were stored under appropriate frozen (−20 °C) conditions until they were shipped to National Center for Environmental Health for testing. The lower limit of detection (LLOD) for vitamin B12 was 30 pg/mL (i.e., 22.14 pmol/L). The coefficient of variation for this assay was lower than 4%^23^.

#### Serum folic acid measurement

Serum folic acid was studied using an automated cell counter and chemiluminescence. A serum folic acid level of less than 4 ng/ml was considered to indicate a folate deficiency (Roche Elecsys 170 System (Roche Diagnostics, Indianapolis, IN).

#### Serum 25OHVD measurement

To measure 25OHVD we used high performance liquid chromatography (HPLC) kits following the manufacturer’s instructions (ImmuChrom GmbH, Heppenheim, Germany. Quantification of 25-OH vitamin D3 was made by HPLC system with UV (264 nm) detector (Thermo Electron, San Jose, CA, USA). The intra-assay coefficient of variation was 0.9–2.9%, and the calculated inter-assay coefficient of variation was 1.7–3.9% and recovery was 91%^24^.

#### Serum magnesium measurement

The Beckman Coulter AU System Magnesium procedure utilizes a direct method in which magnesium forms a colored complex with xylidyl blue in a strongly basic solution, where calcium interference is eliminated by glycoletherdiamine-N,N,N′,N′-tetraacetic acid (GEDTA) (Beckman Coulter, Inc., 250 S. Kraemer Blvd. Brea, CA 92821, USA). The magnesium levels were expressed in milligrams per deciliter (mg/dL). The within run precision for serum samples was less than 1.26% CV and total precision is less than 1.53% CV^25^.

#### Assessment of endothelial dysfunction

The determination of endothelial dysfunction was performed according to the method described by Celemajer *et al*.^26^. Measurements were made by a single observer using an ATL 5000 ultrasound system (Advanced Technology Laboratories Inc., Bothell, WA., USA) with a 12-Mhz probe. All vasoactive medications were withheld for 24 hours before the procedure. The subjects remained at rest in the supine position for at least 15 min before the examination started. Subject’s right arm was comfortably immobilized in the extended position to allow consistent recording of the brachial artery 2–4 cm above the antecubital fossa. Three adjacent measurements of end-diastolic brachial artery diameter were made from single 2-D frames. All ultrasound images were recorded on S-VHS videotape for subsequent blinded analysis. The maximum FMD diameters were calculated as the average of the three consecutive maximum diameter measurements after hyperemia. The FMD levels were then calculated as the percent change in diameter compared with baseline resting diameters.

### End-points

The primary endpoint was FMD percentage change in cohort at the 12^th^ week of the study. Secondary endpoints included status of the anti-oxidant parameters, inflammatory marker (hsCRP), endothelial biomarkers (ADMA, HOMA), and serum lipid profile.

### Statistical Methods

With a study population of 178 patients and a standard deviation of the difference of FMD change after therapies of 0.50, our study has a 90% power to detect as statistically significant with a p value < 0.001 a FMD change of 0.2% or greater. Non-normally distributed variables were expressed as median (range) and normally distributed variables as mean ± SD. A p value < 0.05 was considered to be statistically significant. Kolmogorov Smirnov test was used for analysis distribution of data. One Way ANOVA, student t test and paired sample t test was used for compare numeric data. Comparisons between groups of nominal variables were performed with the Chi-square test. Pearson’s correlation analysis was used to determine correlations between two variables. Multiple regression analysis was applied to identify the independent correlates of flow mediated dilatation. Multiple regression models were built by including all significant univariate correlates of the outcome measures (FMD changes). The models had sufficient power to test the independent association of FMD with relevant correlates, i.e. at least 10 observations per covariate in the same models. All statistical analyses were performed by using SPSS 21.0 (SPSS Inc., Chicago, IL, USA) statistical package^27^.

### Ethical permissions

Clinical trials ethics committee of ministry of health, Ankara, Turkey (2019/188) approved the study protocol and informed consent was obtained from each subject. All methods and procedures in this study were carried out in accordance with relevant guidelines and regulations.

## Results

### Baseline characteristics

The mean age of the patients was 58 ± 14 years and 52.8% were male (n = 94). The chronic disease groups studied were; rheumatoid arthritis (n = 21), familial Mediterranean fever (FMF) (n = 22), diabetes mellitus type-2 (n = 24), hypertension (n = 25), multiple sclerosis (n = 22), Chronic obstructive pulmonary disease (COPD) (n = 22), Alzheimer disease (n = 21), Cancer (n = 21).

### The effect of AAL, AIC and AOL therapies

Table 1 shows the longitudinal changes of selected parameters in the 178 patients that completed the study. Following AAL, AIC and AOL therapies, ADMA, MDA, PTX3, HOMA index, hsCRP, eGFR, SBP, DBP, HbA1c, total cholesterol, triglyceride, and LDL levels were significantly decreased and serum albumin, FMD, CuZn-SOD, GSH-Px, HDL cholesterol, Vitamin B12, 25OHVD and folic acid levels were significantly increased (Table 1). However, the eGFR and BMIs of the patients did not change significantly during the study period.

**Table 1:** Baseline clinical and laboratory characteristics of patients and longitudinal changes following 12 weeks of AAL, AIC and AOL therapies.

	AAL, AIC and AOL therapies (n = 178)		
	Baseline	12^th^ week	p
Age (years)	58±14^a^		
Sex (M/F)	94/84		
BMI (kg/m^2^)	25.3±2.6^a^	26.5±2.3	0.24
Total Cholesterol (mg/dl)	218.9±63.6^a^	173.8±98.1**	<0.001
Triglycerides (mg/dl)	169.4±23.1^a^	138.6±21.5**	<0.001
LDL-cholesterol (mg/dl)	127 (59–166)^b^	91 (55–127)**	<0.001
HDL-cholesterol (mg/dl)	38.6±6.9^a^	44.9±8.1**	<0.001
Systolic blood pressure (SBP) (mmHg)	139±5^a^	129±9**	<0.001
Diastolic blood pressure (DBP) (mmHg)	85 (65–112)^b^	78 (60–91)**	<0.001
eGFR (ml/min/ 1.73 m^2^)	108 (77–131)^b^	106 (79–133)	0.67
Hemoglobin (g/dl)	10.7±1.3^a^	13.2±0.9**	<0.001
25OHVD (nmol/L)	31.1±12.1^a^	62.1±13.6**	<0.001
Vitamin B_12_ (pg/ml)	209.2±72.1^a^	402.9±113.9**	<0.001
Folic acid (ng/ml)	6.2±2.4^a^	9.8±2.2**	<0.001
Serum Magnesium (mg/dl)	2.1±0.4^a^	3.2±0.7**	<0.001
HOMA-IR	3.3±0.4^a^	1.4±0.6**	<0.001
Serum albumin (g/dl)	3.6±0.2^a^	4.1±0.3**	0.02
HbA1c (%)	7.02±1.1^a^	5.8±0.8**	<0.001
Malondialdehyde(MDA) (nmol/ml)	5.9±2.2^a^	2.2±0.6**	<0.001
CuZn-SOD (U/ml)	336.5±138.1^a^	505.1±114.6**	<0.001
GSH-Px (U/ml)	47.3±15.3^a^	72.6±20.8**	<0.001
hs-CRP (mg/l)	25 (3–111)^b^	3 (2–11)**	<0.001
PTX3 (ng/ml)	11.4 (1.8–92.4)^b^	2.2 (1.1–16.3)**	<0.001
ADMA(µmol/l)	4.6±1.9^a^	1.3±0.6**	<0.001
FMD (%)	5.2 (4.0–7.2)^b^	6.5 (4.7–8.8)**	<0.001

BMI, Body mass index; BP, blood pressure; 25OHVD, 25 hydroxy-vitamin D ; FMD, endothelium dependent vasodilatation;

ADMA: Asymmetric dimethyl arginine; HOMA, homeostasis model assessment; iPTH, intact parathyroid hormone,

eGFR, estimated glomerular filtration rate; hsCRP, high sensitivity C reactive protein; PTX3, pentraxin 3;

CuZn-SOD, copper zinc-superoxide dismutase; GSH-Px, glutathione peroxidase; MDA, Malondialdehyde

Morinda citrifolia (anti-atherosclerotic liquid- AAL) (3 ml once per day)

Omega-3 (anti-inflammatory capsules- AIC) (3 capsules once per day)

Extract with Alaskan blueberry and 21 different red purple fruit vegetables (anti-oxidant liquid- AOL) (30 ml once per day)

^a^mean(standard deviation, ^b^median (interquartile range)

**Paired Samples t-test, statistically significant (p < 0.05) compared with treatment group (Before and after treatment)

Data are means ± SD and median. NS, not significant

### Univariate correlations

At baseline, FMD was negatively correlated with serum ADMA, MDA, PTX3, HOMA SBP, DBP, and hsCRP levels and positively correlated CuZn-SOD, GSH-Px, Hemoglobin and eGFR levels (Table 2). The negative correlations between FMD levels and ADMA, MDA, PTX3, HOMA, SBP, DBP, and hsCRP were present after the 12-week therapy period as well (Table 3). The positive correlations between FMD levels and CuZn-SOD and eGFR were also present after the 12-week therapy period as well (Table 3). The increase (%) in FMD was negatively correlated with the reduction (%) in serum ADMA, MDA, PTX3, hsCRP and HOMA concentrations (rho = −0.63, p < 0.001; rho = −0.58, p < 0.001; rho = −0.49, p < 0.001; rho = −0.43, p < 0.001, respectively) (Table 4). The increase (%) in FMD was positively correlated with the elevation (%) in CuZn-SOD concentration (rho = 0.48, p < 0.001) (Table 4).

**Table 2:** Analysis of association between FMD and some relevant parameters by univariate and multivariate linear regression in baseline.

	Univariate Rho (p)	Multivariate Beta (p)
*Baseline data (r*^2^ = *0.74)*		
ADMA (µmol/l)	−0.78 (<0.001)	−0.38 (<0.001)
Malondialdehyde (MDA) (nmol/ml)	−0.68 (<0.001)	NS
CuZn-SOD (U/ml)	0.66 (<0.001)	0.13 (0.01)
GSH-Px (U/ml)	0.32 (<0.001)	NS
PTX3 (ng/ml)	−0.61 (<0.001)	−0.14 (0.01)
hs-CRP (mg/l)	−0.72 (0.02)	NS
eGFR (ml/min/ 1.73 m^2^)	0.23 (0.001)	NS
Hemoglobin (g/dl)	0.59 (<0.001)	0.33 (<0.001)
Total Cholesterol (mg/dl)	−0.51 (<0.001)	NS
Triglyceride (mg/dl)	−0.17 (<0.001)	NS
LDL cholesterol (mg/dl)	−0.27 (<0.001)	NS
HDL cholesterol (mg/dl)	0.13 (0.05)	NS
Systolic blood pressure (mmHg)	−0.59 (<0.001)	−0.13 (0.004)
Diastolic blood pressure (mmHg)	−0.27 (<0.001)	NS
Serum albumin (g/dl)	0.08 (0.23)	NS
HOMA-IR	−0.29 (<0.001)	NS
HbA1c (%)	−0.54 (<0.001)	NS

ADMA: Asymmetric dimethyl arginine; HOMA, homeostasis model assessment; eGFR, estimated glomerular filtration rate; hsCRP, high sensitivity C reactive protein; PTX3, pentraxin 3; CuZn-SOD, copper zinc-superoxide dismutase; GSH-Px, glutathione peroxidase; MDA, Malondialdehyde.

**Table 3:** Analysis of association between FMD and some relevant parameters by univariate and multivariate linear regression after 12 weeks of AAL, AIC and AOL therapies.

	Univariate Rho (p)	Multivariate Beta (p)
*Baseline data (r*^2^ = *0.63)*		
ADMA (µmol/l)	−0.68 (<0.001)	−0.35 (<0.001)
Malondialdehyde (MDA) (nmol/ml)	−0.69 (<0.001)	−0.30 (<0.001)
CuZn-SOD (U/ml)	0.42 (<0.001)	NS
GSH-Px (U/ml)	0.11 (0.09)	NS
PTX3 (ng/ml)	−0.34 (<0.001)	−0.12 (0.007)
hs-CRP (mg/l)	−0.38 (<0.001)	−0.21 (<0.001)
eGFR (ml/min/ 1.73 m^2^)	0.35 (<0.001)	0.14 (0.002)
Hemoglobin (g/dl)	0.12 (0.06)	NS
Total Cholesterol (mg/dl)	−0.11 (0.09)	NS
Triglyceride (mg/dl)	−0.38 (<0.001)	NS
LDL cholesterol (mg/dl)	−0.26 (<0.001)	NS
HDL cholesterol (mg/dl)	0.09 (0.25)	NS
Systolic blood pressure (mmHg)	−0.15 (0.04)	NS
Diastolic blood pressure (mmHg)	−0.20 (0.004)	−0.12 (0.004)
Serum albumin (g/dl)	0.09 (0.20)	NS
HOMA-IR	−0.32 (<0.001)	NS

ADMA: Asymmetric dimethyl arginine; HOMA, homeostasis model assessment; eGFR, estimated glomerular filtration rate; hsCRP, high sensitivity C reactive protein; PTX3, pentraxin 3; CuZn-SOD, copper zinc-superoxide dismutase; GSH-Px, glutathione peroxidase; MDA, Malondialdehyde.

**Table 4:** Analysis of association between change (∆) in FMD and relevant parameters by univariate and multivariate linear regression analysis.

	Univariate Rho (p)	Multivariate Beta (p)
∆ FMD (%) *(r*^2^ = *0.30)*		
Change in ADMA (µmol/l)	−0.63 (<0.001)	−0.25 (0.01)
Change in MDA (nmol/ml)	−0.58 (<0.001)	−0.18 (0.02)
Change in CuZn-SOD (U/ml)	0.48 (<0.001)	NS
Change in GSH (U/ml)	0.02 (0.75)	NS
Change in HOMA	−0.21 (0.001)	NS
Change in eGFR (ml/min/ 1.73 m^2^)	−0.03 (0.62)	NS
Change in hsCRP (mg/l)	−0.45 (<0.001)	NS
Change in PTX3 (ng/ml)	−0.49 (<0.001)	−0.21 (0.01)
Change in SBP (mmHg)	−0.26 (<0.001)	NS
Change in DBP (mmHg)	−0.11 (0.12)	NS
Change in Hemoglobin (g/dl)	0.07 (0.32)	NS
Change in Total Cholesterol (mg/dl)	−0.05 (0.49)	NS
Change in Triglyceride (mg/dl)	−0.11 (0.12)	NS
Change in LDL Cholesterol (mg/dl)	−0.12 (0.07)	NS
Change in HDL Cholesterol (mg/dl)	0.02 (0.82)	NS
Change in HbA1c (%)	−0.26 (<0.001)	NS

ADMA: Asymmetric dimethyl arginine; HOMA, homeostasis model assessment; eGFR, estimated glomerular filtration rate; hsCRP, high sensitivity C reactive protein; PTX3, pentraxin 3; CuZn-SOD, copper zinc-superoxide dismutase; GSH-Px, glutathione peroxidase; MDA, Malondialdehyde.

### Multivariate regression analysis

**Figure 2:**
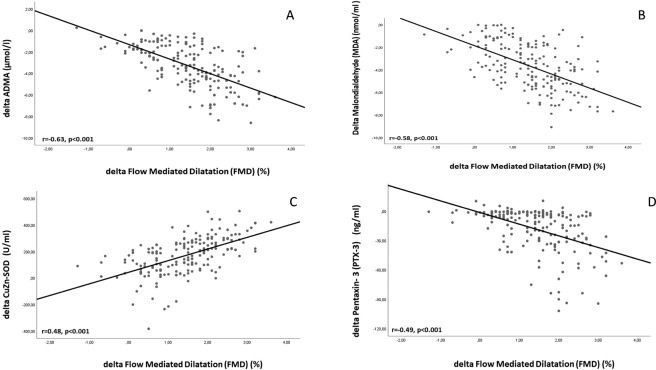
Scatter-plot graphs between FMD and ADMA, MDA, CuZn-SOD, PTX-3. (**A**) The scatter plot showing the significant negative relationship between the changes (%) in ADMA and FMD during the 12-week AAL, AIC and AOL interventions. (**B)** The scatter plot showing the significant negative relationship between the changes (%) in MDA and FMD during the 12-week AAL, AIC and AOL interventions. **(C)** The scatter plot showing the significant negative relationship between the changes (%) in CuZn-SOD and FMD during the 12-week AAL, AIC and AOL interventions. **(D)** The scatter plot showing the significant negative relationship between the changes (%) in PTX-3 and FMD during the 12-week AAL, AIC and AOL interventions.

We next investigated the independence of the observed correlations (Table 2) with FMD using a multiple linear regression model incorporating sex and age as well as variables significantly associated with FMD (ADMA, MDA, CuZn-SOD, GSH-Px eGFR, PTX3, SBP, DBP, hemoglobin, serum albumin, HOMA-IR, HbA1c, and hs-CRP). Briefly, FMD levels were independently related to ADMA, CuZn-SOD Hemoglobin, SBP and PTX3 before noni, omega-3 and anti-oxidant therapies (Table 2). Additionally, FMD levels were also independently related to ADMA, MDA, hsCRP, eGFR, DBP and PTX3 after AAL, AIC and AOL therapies (Table 3). The models included changes in 24-hour protein excretion, eGFR, SBP, DBP, serum albumin and hs-CRP, as well as change in FMD or PTX3, respectively. Briefly, change in serum FMD levels was only independently related to changes in ADMA (p = 0.01), MDA (p = 0.02) and PTX3 (p = 0.01) (Fig. 2).

## Discussion

This is the first study to demonstrate important and effective the correction of 3 important pathways in the occurrence of chronic diseases using 3 different products. In the present study, we report the results of an open-label 12-week trial investigating the impact of initiation of AAL, AIC and AOL therapies in chronic diseases. We found that the expected improvement in endothelial dysfunction was accompanied by a significant reduction in the inflammatory markers hsCRP and PTX3, oxidative stress marker MDA and anti-atherosclerotic marker ADMA, the change in which both correlated independently with an observed improvement in ultrasonographically measured FMD. With three-month use of these three products (AAL, AIC and AOL) vitamin B12, vitamin D, folic acid, hemoglobin, HDL cholesterol, serum albumin and magnesium levels were statistically significantly increased, while total cholesterol, triglyceride, LDL cholesterol, HOMA, HbA1c, SBP and DBP values were significantly decreased.

This is of long-term relevance for patients with respect to the potential impact of inflammation. Inflammation can lead to the development of chronic diseases as well as enhance oxidative stress and endothelial dysfunction, both of which are thought to be key steps in the development of chronic diseases. This impact is illustrated by a natural experiment across two diverse populations. Alaskan Eskimos/Inuit has the lowest rate of mortality due to ischemic heart disease among the white populations across the US. It was shown that these people has high omega-3 fatty acids levels related with huge amount of salmon consumption^28,29^. Omega-3 fatty acids possibly use the mechanisms to reduce cardiovascular risk factors such antithrombotic and anti-inflammatory effects. With this way it increases endothelial cell function which is mainly in the vessels, and also reducing adhesion molecules at the same cells. These alterations may help to normalize arterial blood pressure^30^. Clinical trials with DHA-rich oil suggest comparable efficacies to fish oil for protection from cardiovascular risk factors by lowering plasma triglycerides, and inflammation^11,12^. We have shown for the first time in the literature that the use of these three products, and especially the effect of Omega 3, with known anti-inflammatory properties. The literature has suggested this in that Omega 3 appears to significantly lower the inflammatory markers hsCRP and PTX3.

The discrepancy between oxidant and antioxidant products cause oxidative stress which is the result of increased production of free radicals and reactive oxygen species. At the end this process leads to destruct cellular functions^3^. Berries are one of the best-known sources of antioxidants and can be supplemented by diet. There are several types of berries which contains a variety of bioactive nutrients such as phenolic compounds, flavonoids, and tannins. Individually or synergistically, these have been shown to reduce the risk of several disorders. Mounting evidence suggests that consumption of berries confer antioxidant and anticancer protection to humans and animals by a number of mechanisms such as free radical scavenging, protection from DNA damage, induction of apoptosis and inhibition of cellular growth and proliferation of cancer cells^31,32^. Previous research has suggested that blueberry consumption may attenuate these processes^33,34^. We have shown for the first time in the literature that the oxidative stress markers MDA decreased and CuZn-SOD and GSH-Px can be increased by use of these three products especially blueberry and 21 red purple fruit and vegetable effects known for anti-oxidant properties.

Endothelial dysfunction, the impairment of regulatory functions of the endothelium for vasodilatation, smooth muscle cell proliferation, and fibrinolysis, is pivotal in the pathogenesis of cardiovascular disease. In a review of *Morinda citrifolia*, associations with many chronic diseases have been identified. Noni juice (*Morinda citrifolia*) is a globally popular health beverage originating in the tropics. Human clinical trials are necessary for a precise understanding of what the health benefits of noni juice might be. Human intervention studies also indicate that noni juice may improve joint health, increase physical endurance, increase immune activity, Blood Lipid Normalization, hs-CRP and Homocysteine Reduction, Control of Advanced Glycation End Products (AGEs) and Glycosylated Hemoglobin, inhibit glycation of proteins, aid weight management, help maintain bone health in women, help maintain cancer and rheumatoid arthritis, help maintain normal blood pressure and Mitigation of Osteoporosis, and Gingivitis and improve gum health^35–48^. It has shown for the first time in the literature that serum ADMA levels from endothelial dysfunction markers have decreased and FMD has increased with the use of these three products, and especially with the effect of noni fruit, which is known for its anti-atherosclerotic properties.

The limitations of our study must be mentioned. First, the number of the patients was small and this limits the power to detect changes in a marker as variables as ADMA, MDA and PTX3. In our study, we included patients with different chronic diseases. Care was taken to ensure that the drugs used by the patients included in the study were not among the drugs that could affect endothelial function. However, the fact of different diseases and different age ranges were another limitation of our study. Finally, as we evaluated a much-selected group of different many chronic diseases this does not represent potentially much more heterogeneous patients with chronic disease population at large and as such these results need confirmation in other studies.

In conclusion, we found that 12 weeks of AAL, AIC and AOL therapies significantly reduced serum ADMA, MDA and PTX3 levels in direct proportion to the observed improvement in endothelial functions in patients with chronic diseases. ADMA, MDA and PTX-3 were the markers responsible for the improvement of endothelial dysfunction in patients using these three products with anti-inflammatory, anti-oxidant and anti-atherosclerotic properties. In this study, it has been shown for the first time in the literature that endothelial function is improved by correcting these three important pathways responsible for disease formation mechanisms with 3 different food supplements.
